# Anuran accents: Continental‐scale citizen science data reveal spatial and temporal patterns of call variability

**DOI:** 10.1002/ece3.6833

**Published:** 2020-10-06

**Authors:** Savannah J. Weaver, Corey T. Callaghan, Jodi J. L. Rowley

**Affiliations:** ^1^ Australian Museum Research Institute Australian Museum Sydney NSW Australia; ^2^ Department of Biology Bucknell University Lewisburg PA USA; ^3^ Centre for Ecosystem Science School of Biological, Earth and Environmental Sciences UNSW Sydney Sydney NSW Australia; ^4^ Ecology & Evolution Research Centre School of Biological, Earth and Environmental Sciences UNSW Sydney Sydney NSW Australia

**Keywords:** advertisement call, bioacoustics, frog, geographic variation, isolation by distance, temporal variation

## Abstract

Many animals rely on vocal communication for mating advertisement, territorial displays, and warning calls. Advertisement calls are species‐specific, serve as a premating isolation mechanism, and reinforce species boundaries. Nevertheless, there is a great deal of interspecific variability of advertisement calls. Quantifying the variability of calls among individuals within a species and across species is critical to understand call evolution and species boundaries, and may build a foundation for further research in animal communication. However, collecting a large volume of advertisement call recordings across a large geographic area has traditionally posed a logistical barrier. We used data from the continental‐scale citizen science project FrogID to investigate the spatial and temporal patterns of call characteristics in six Australian frog species. We found intraspecific call variability in both call duration and peak frequency across species. Using resampling methods, we show that variability in call duration and peak frequency was related to the number of individuals recorded, the geographic area encompassed by those individuals, and the intra‐annual time difference between those recordings. We conclude that in order to accurately understand frog advertisement call variation, or “anuran accents,” the number of individuals in a sample must be numerous (*N* ≥ 20), encompass a large geographic area relative to a species' range, and be collected throughout a species' calling season.

## Graphical abstract

Quantifying the variability in a species' advertisement vocalization is critical to understand call evolution and species boundaries, and may build a foundation for further research on vocalizations. We used data from the continental‐scale citizen science project FrogID to investigate the spatial and temporal patterns of call characteristics in six Australian frog species. We conclude that in order to accurately understand frog advertisement call variation, or “anuran accents,” the number of individuals in a sample must be numerous (*N* ≥ 20), encompass a large geographic area relative to the species’ range, and be collected throughout the species' calling season. Photo: *Litoria chloris*.

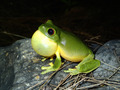

## 1 INTRODUCTION

Vocal communication is widely used throughout the animal kingdom, including for mating advertisement, territorial displays, and warning calls (Brumm, 2013; Gerhardt & Huber, 2002). Advertisement vocalizations are made by males to attract females (Eriksson & Wallin, 1986; Ryan, 2001; Wells, 1977), and females use call characteristics like pitch, volume, and complexity to choose the best mate (Boul et al., 2007; Pedroso et al., 2013). Many taxa, namely birds (Ballentine, 2004; Clark et al., 2006), frogs (Ryan, 2001; Stebbins & Cohen, 1997; Wells, 2007), and insects (Gerhardt & Huber, 2002; Saarikettu et al., 2005), rely on vocalizations to find and secure mates for reproduction. Because these advertisement calls are species‐specific (Curé et al., 2012; Searcy et al., 1981) and serve as a premating isolation mechanism (Littlejohn, 1969), they reinforce species boundaries (Braune et al., 2008; Pedroso et al., 2013).

Advertisement vocalizations best serve their purpose by being unique to, and consistent within, a species. Yet we consistently find intraspecific variation across animals with species‐specific vocalizations. For example, nonhuman primates (Marler, 1973; Mitani et al., 1999), insects (Zuk et al., 2001), marine mammals (Cerchio et al., 2001; Rendell & Whitehead, 2005; Terhune et al., 2001), and birds (González & Ornelas, 2014; Haftorn & Hailman, 1997; Handford, 1988) all exhibit varying levels of intraspecific variation in vocal communication. Despite a breadth of research on numerous taxa, a clear ecological pattern for intraspecific variability of vocalizations remains enigmatic. Research has investigated spatial and temporal trends of vocalizations, but results from previous studies are inconsistent. For example, local variability of a species‐specific call can change over time (Adret‐Hausberger, 1986; Ince et al., 1980) or stay consistent without measurable temporal variation (Fournet et al., 2018; Whitney, 1992). There is also evidence that vocalization differences can be predicted by geographic distances (Marova et al., 2010; Röhr et al., 2020; Searfoss et al., 2020) or barriers (Jang et al., 2011; Zuk et al., 2001), but we do not know whether geographic isolation is a driver or product of different vocalizations.

Frogs are a useful taxonomic group to test ecological patterns in intraspecific variability because the advertisement call is a species‐specific trait (Oldham & Gerhardt, 1975), serves as a premating isolation mechanism (Boul et al., 2007; Capranica et al., 1973; Littlejohn, 1969), and is used by researchers to distinguish between and describe new species (Davies et al., 1986; Hoskin, 2007; Rowley et al., 2016; Sullivan et al., 1996). Additionally, the developmental biology of most frog species eliminates the possibility of learning as a confounding variable in studies of their vocalizations (Duellman & Trueb, 1994; Wells, 2007), allowing more confidence in a genetic basis for frog call characteristics (Welch et al., 2014), and a more direct connection to phylogeny (Bosch & De la Riva, 2004; Erdtmann & Amézquita, 2009; Ryan & Wilczynski, 1991).

Although species‐specific, frog advertisement calls do vary among individuals and populations, and even within individuals (Bee et al., 2001, 2010; Gambale et al., 2014; Gerhardt, 1991; Gerhardt & Huber, 2002; Hernández‐Herrera & Pérez‐Mendoza, 2020; Pettitt et al., 2013). Factors that correlate with population‐level variation of frog calls include habitat, discrete populations, and geographic isolation by distance (Jang et al., 2011; Littlejohn & Roberts, 1975; Ohmer et al., 2009; Rafiński & Babik, 2000; Rodríguez et al., 2010; Ryan & Wilczynski, 1991), although geographic patterns are not always found (Baraquet et al., 2015; Giacoma et al., 1997). Intra‐annual time difference (measured as difference in days within a species‐specific breeding season) may play a role, but few studies have tested this in frogs (see Gambale et al., 2014; Giacoma et al., 1997; Smith & Hunter, 2005). At the individual level, calls also vary in relation to body size and temperature (Blair, 1964; Kasuya & Shiobara, 1996; Rodríguez et al., 2010; Sullivan & Hinshaw, 1990). However, not all properties covary significantly or consistently, and residual variation remains after body size and temperature effects are accounted for (Castellano et al., 2000; Jang et al., 2011). Quantifying the variability of calls among individuals within a species and across species is critical to understand call evolution and species boundaries, as well as to inform methods for future research on frog vocalizations.

To date, studies of frog advertisement call variability have been limited in geographic, taxonomic, and temporal extent, largely as a result of the logistical challenges in collecting the quantity and quality of data required to investigate macroecological trends. Recently, however, the rise of citizen science projects has enabled data collection on a much greater temporal and spatial scale than ever before (Aceves‐Bueno et al., 2017; Callaghan et al., 2019; Lukyanenko et al., 2016; McKinley et al., 2017; Silvertown, 2009). Such datasets facilitate a breadth of ecological studies that would not be feasible otherwise (Diblíková et al., 2019; Mitchell et al., 2020; Searfoss et al., 2020).

We use continental‐scale citizen science data collected and submitted through the FrogID project to investigate spatial and temporal patterns of call characteristics in six Australian frog species. We hypothesized that within each species, call characteristics would vary across geographic area and breeding season, with both spatial and temporal variability following an isolation by distance/difference model. We also hypothesized that a larger geographic range or larger maximum body size would enable greater vocal variability within a species.

## 2 METHODS

### 2.1 FrogID

FrogID (www.frogid.net.au) is a citizen science project led by the Australian Museum where volunteers use their smartphones to record calling frogs. All submissions are validated by experts at the Australian Museum (Rowley & Callaghan, 2020; Rowley et al., 2019). To date, FrogID has received over 150,000 submissions, resulting in approximately 220,000 records of calling frogs, including 198 of Australia's 240 known frog species.

### 2.2 Study species

**FIGURE 1:**
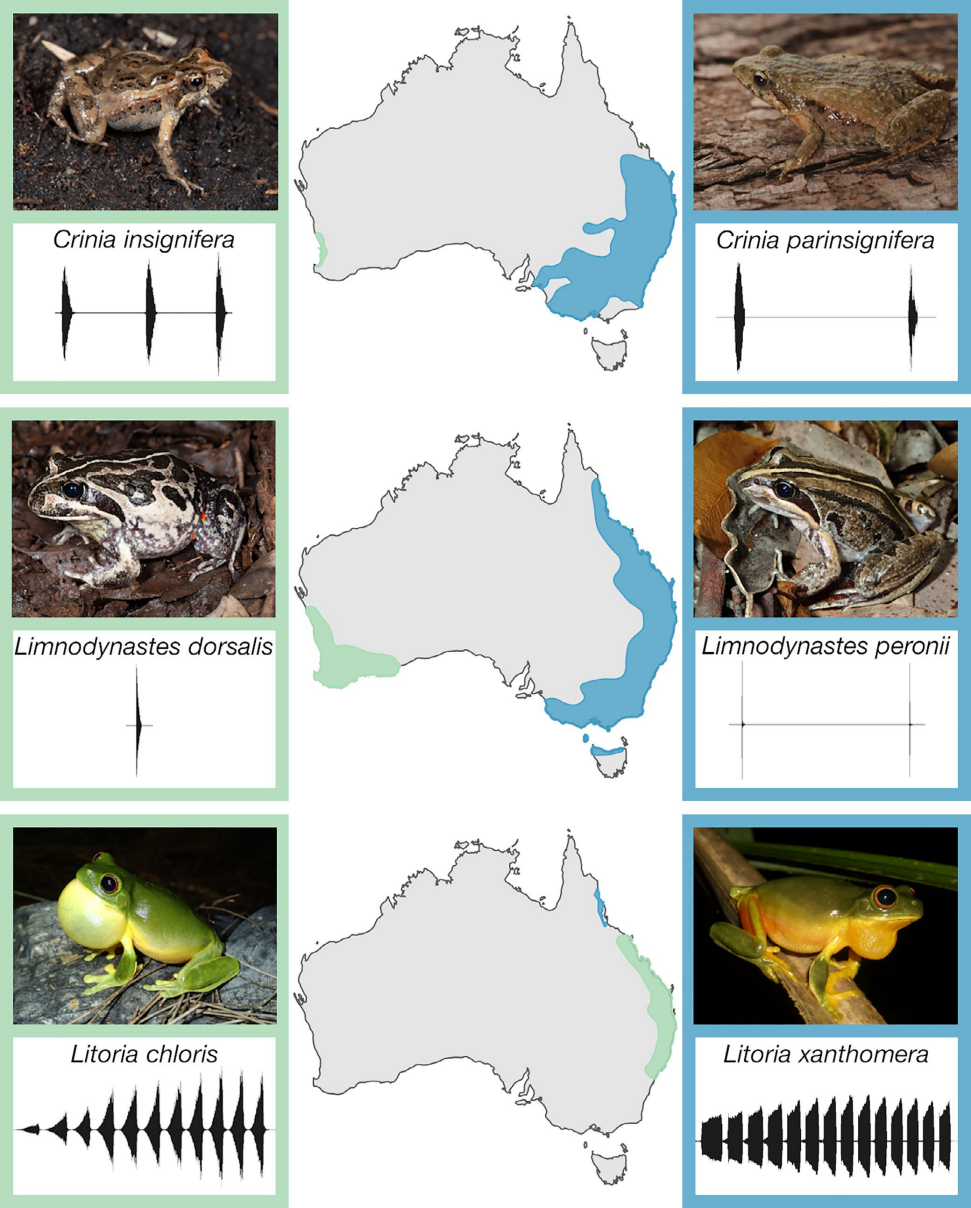
Study species, geographic range, and spectrograms of representative advertisement calls. Relative amplitudes over time are as follows: *Crinia insignifera*, 3 calls: ±500, 7 s; *Crinia parinsignifera*, 2 calls: ±500, 6 s; *Limnodynastes dorsalis*, 1 call: ±20, 2 s; *Limnodynastes peronii*, 2 calls: ±6, 8 s; *Litoria chloris*, 1 call, 11 notes: ±1, 8 s; *Litoria xanthomera*, 1 call, 14 notes: ±25, 15 s. Photographs by Jodi Rowley (*C*. *parinsignifera*, *Lim*. *peronii*, *Lit*. *chloris*, *Lit*. *xanthomera*) and Stephen Mahoney (*C*. *insignifera*, *Lim*. *dorsalis*)

We selected *Crinia insignifera*, *Crinia parinsignifera*, *Limnodynastes dorsalis*, *Limnodynastes peronii*, *Litoria chloris*, and *Litoria xanthomera* to be the focus of our study (Figure 1). Each has a high number of FrogID submissions, distributed throughout their geographic range. These three congeneric pairs of species are phylogenetically closely related and have similar male advertisement calls, but are allopatric, and have different geographic range sizes.

### 2.3 Call selection and analysis

Increasingly, descriptive studies of frog calls are using smartphone recordings (Modak et al., 2016; Roh et al., 2014). The audio clarity and quality of FrogID recordings vary, resulting from differences in smartphones used, the distance between the observer and the calling frog, and the amount of background noise, including other calling frogs, in the recording (Rowley & Callaghan, 2020; Rowley et al., 2019). Although different phone models vary in their detection range and frequency response (Zilli, 2015), almost all smartphone models have a flat frequency response up to a threshold (Kardous & Shaw, 2014). Recordings made with the FrogID app are saved as MPEG AAC audio files, a form of audio compression that affects all frequencies uniformly (Brandenburg, 1999). While frequencies above 17 kHz are not represented in FrogID recordings (Rowley et al., 2019), all known Australian frogs have advertisement calls below 10kHz (Loftus‐Hills, 1973; Rowley et al., unpublished data). The dominant frequency of the advertisement calls of frog species in this study was all below 6 kHz.

We used FrogID recordings verified up to October 2019 and filtered for those in which only the target species was calling, increasing the likelihood that a recording would be of sufficient quality for analysis. We then filtered by locality to maintain spatial representation in those species with large numbers of FrogID recordings. For *C*. *insignifera*, *C. parinsignifera*, and *Lim*. *dorsalis*, we only used one recording from a given latitude and longitude combination to avoid unintentionally using several recordings of the same individual. For *Lim*. *peronii*, a species with a large geographic range and numerous FrogID recordings, we only used recordings collected at least 1 km apart from each other. We did not eliminate any recordings based on location for *Lit*. *chloris* and *Lit. xanthomera* because fewer FrogID recordings were available. This process of geographic filtering nullifies questions of temporal change at the same location or of the same individual, but we were more interested in among individual, rather than within individual, macroecological trends. After filtering by locality, we excluded recordings that were of insufficient quality (too many overlapping calls to pick out a single individual or too faint) or duration (not capturing enough calls of the individual). Table 1 lists number of recordings of each species before and after filtering.

**TABLE 1:** Range size, maximum male snout–vent length (SVL), recording details, and call values for each species

Species	Range size (km^2^)	Maximum Male SVL (mm)	Earliest recorded individual	Latest recorded individual	Recordings evaluated	Recordings analyzed (%)	Mean call duration ± *SD* (s)	Mean peak frequency ± *SD* (Hz)
*Crinia insignifera*	16,853	25	4‐May‐18	30‐Aug‐19	226	123 (54)	0.22 ± 0.06	3,353 ± 437
*Crinia parinsignifera*	1,116,599	22	10‐Nov‐17	8‐Sep‐19	383	221 (58)	0.27 ± 0.13	3,746 ± 400
*Limnodynastes dorsalis*	311,818	64	24‐Nov‐17	14‐Sep‐19	181	82 (45)	0.16 ± 0.07	609 ± 130
*Limnodynastes peronii*	1,071,422	69	4‐Nov‐17	20‐Sep‐19	487	232 (48)	0.12 ± 0.07	1,033 ± 327
*Litoria chloris*	276,209	62	13‐Nov‐17	8‐Mar‐19	108	48 (44)	0.97 ± 0.19	1873 ± 190
*Litoria xanthomera*	14,196	56	21‐Nov‐17	15‐May‐19	102	56 (55)	0.75 ± 0.14	2,160 ± 198

We define a call as the entire assemblage of acoustic signals for a given vocalization and a note as an individual unit of sound, following definitions presented by Duellman and Trueb (1986). For *C*. *insignifera*, *C*. *parinsignifera*, *Lim*. *dorsalis*, and *Lim*. *peronii*, each call was a single note, and for *Lit*. *chloris* and *Lit*. *xanthomera*, each call consisted of several notes. For consistency across species, we analyzed the notes of the species in the genus *Litoria* as calls. FrogID recordings were converted into WAV format at a 48 kHz sampling rate and 16 bits per sample. We used Raven Sound Software (Pro Version 1.5, Cornell Lab of Ornithology) to visualize waveform and spectrogram for each recording. We set the spectrogram window size to 512 with 50% overlap and band‐filtered all recordings to reduce background noise. For one individual frog in each recording, we measured call duration and peak frequency, which are well‐established call characteristics to analyze (Gergus et al., 1997; Giacoma et al., 1997; Köhler et al., 2017; Littlejohn & Roberts, 1975; Mitchell et al., 2020; Penna & Veloso, 1990). Although we cannot ensure every recording is of a unique individual, it is likely the case, especially for the species which were filtered by locality.

For each recording, we selected 3–10 consecutive advertisement calls of a single individual, a sample size consistent with previous acoustic research (Bionda et al., 2008; Penna & Veloso, 1990). For *Lit*. *chloris* and *Lit*. *xanthomera*, we used all consecutive notes in a single call. For most individuals, we used the first 10 calls recorded to minimize bias. If quality was an issue, we selected the largest possible group of good quality consecutive notes. Quality was based on interference (i.e., wind, human activity, and insects), with preference given to the loudest individual with the most calls recorded. We only included advertisement calls in our analyses because it is the most commonly heard and most taxonomically informative (Köhler et al., 2017). Non‐advertisement calls are less frequent and vary with social context (Perrill & Bee, 1996; Ryan & Wilczynski, 1991; Sullivan & Wagner, 1988).

### 2.4 Statistical analysis

Each individual represents a unique recording location and date. Individual call duration and peak frequency were measured as means of all calls analyzed for each individual, as we were interested in variation among individuals of a species, rather than within individuals. We used resampling approaches to investigate how the intraspecific variability in call duration and peak frequency was influenced by the number of individuals, geographic area covered, and intra‐annual time difference of individuals in a sample of a population.

To test the effect of sample size (i.e., the number of individuals measured as a representative sample of the population), we drew random samples of *N* = 2, 3, 4, … 48 individuals. We used 48 as the maximum number of individuals in a sample because that was the lowest number of individuals we measured for a single species (*Lit*. *chloris*; Table 1). For each sample size, N, we resampled individuals 1,000 times with replacement and calculated the standard deviations of call duration and peak frequency for each sample. We drew qualitative conclusions about the resulting pattern using visual representation (Figure 3).

To test the effect of geographic area and intra‐annual time difference on call variability, we randomly sampled 20 calls with replacement 1,000 times for each species. We calculated the following parameters for each random sample: (a) standard deviation of call duration; (b) standard deviation of peak frequency; (c) geographic area, measured as the convex hull area based on each individual's recording location; and (d) intra‐annual time difference between calls, measured as number of days between the first and last individuals recorded within a breeding season. For species in the genus *Litoria* that have breeding seasons spanning across the calendar year, we relativized the time of year based on the first observations of the breeding season recorded. We set *N* = 20 because it roughly corresponded to the number of individuals necessary for a sample to achieve reliable estimates of variation for a species (Figure 3), and it was approximately half the number of individuals measured for the species with the least individuals (*Lit*. *chloris*; Table 1), allowing for sufficient probability of resampling. We tested other sample sizes (*N* = 10, 30, 40), but found no qualitative differences in our key results, thus only present results for *N* = 20.

We used intraspecific *z*‐scores of each variable to fit individual models for each species (*N* = 6) and to fit a model encompassing the variability across our study species, for four unique comparisons: (a) call duration as a function of intra‐annual time difference; (b) call duration as a function of geographic area; (c) peak frequency as a function of intra‐annual time difference; and (d) peak frequency as a function of geographic area. First, for each comparison, we fit a linear mixed model with species as a random effect to test for the overall influence of the response variable on the predictor variable across species. Second, we fit linear models for each of the six species (i.e., 24 models). Lastly, we fit another linear model to investigate whether the model estimates (i.e., correlation coefficients) extracted from the models for each relationship for each species were associated with range size or body size. Range sizes were extracted from FrogID distribution maps (www.frogid.net.au), which were originally calculated based on species records in the Atlas of Living Australia (www.ala.org.au) and modified by expert opinion. Maximum male body sizes, measured in mm as snout–vent length (SVL), were taken from Anstis (2017).

Temporal aspects of calls often vary with temperature (Bionda et al., 2008; Gambale et al., 2014; Rodríguez et al., 2010; Sullivan & Hinshaw, 1990). Therefore, we tested a generalized linear model for individual call variables across species as a function of temperature, using temperature estimates based on methods from Mitchell et al. (2020). We found that call duration was nonsignificantly positively correlated with temperature (GLM, estimate = .0031, *SE* = .001752, *t* = 1.773, *p*‐value =.077). Typically, temperature shows a direct correlation with temporal call properties such as call duration because as ectotherms, frogs' vocal speed ability is determined by ambient air temperature (Ryan, 2001). However, we found no such correlation. Conversely, peak frequency was significantly negatively correlated (GLM, estimate = −61, *SE* = 8.52, *t* = −7.16, *p*‐value < .0001), even though spectral properties are not usually correlated with temperature, and usually rely more on body size. We acknowledge that temperature effects exist at the individual level, but these models suggest that including temperature in our analyses of call variability would be uninformative. Based on the nonsignificant and counterintuitive trends we found, we chose not to include temperature in our statistical analyses. Further, our resampling approach samples many individuals (*N* = 1,000) at many potential temperatures, so temperature effects are unlikely to influence our macroecological results. Our goal was to encompass the potential range of environmental conditions, rather than to eliminate them. Humidity may also affect body condition generally, but would only affect vocalizations indirectly (Köhler et al., 2017), so we do not consider humidity.

## 3 RESULTS

**FIGURE 2:**
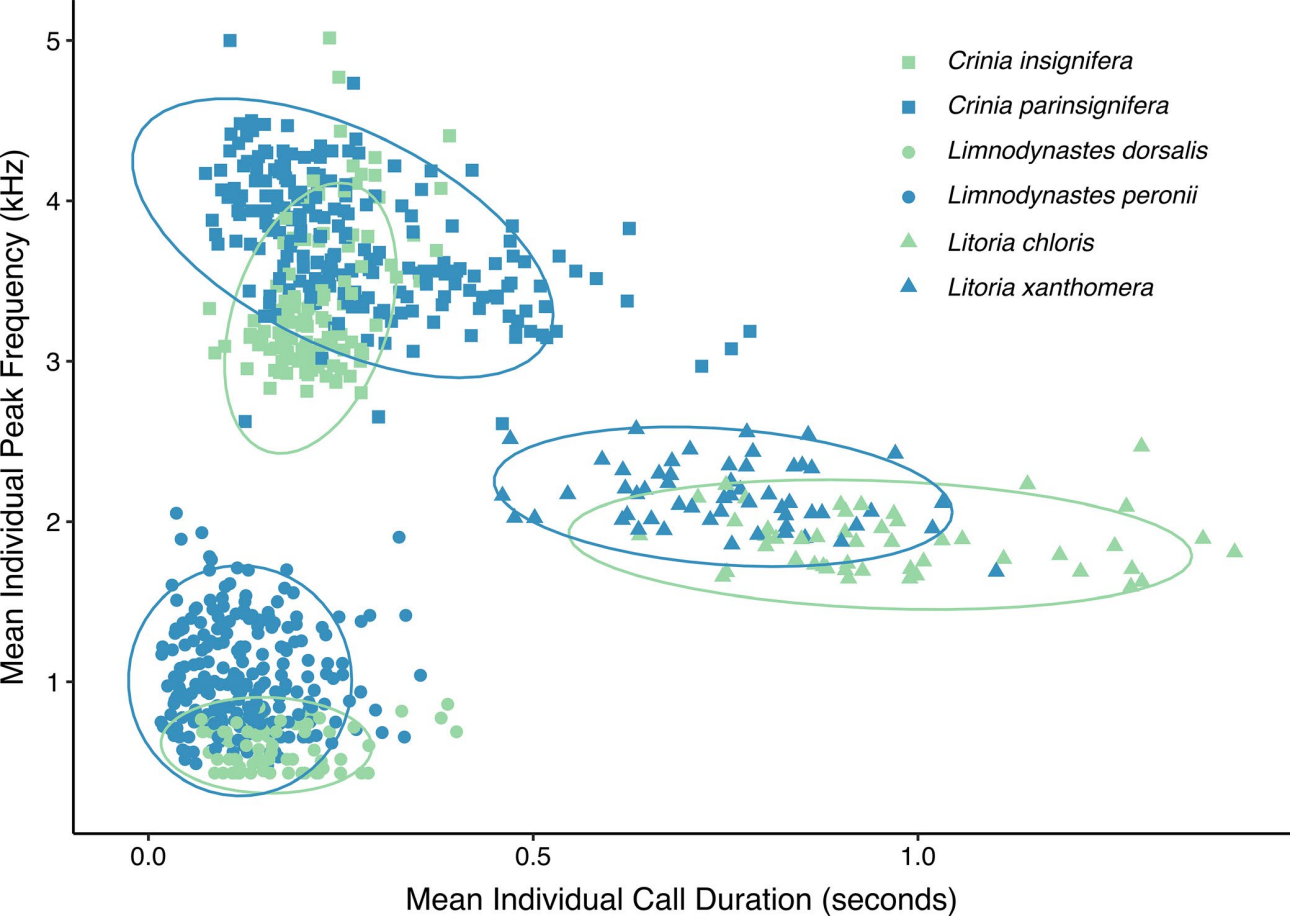
Call duration and peak frequency, measured as the mean value for each individual analyzed, clustered by genus and species. Shapes denote genera: *Crinia* as squares, *Limnodynastes* as circles, and *Litoria* as triangles. Colors distinguish species within each genus: green versus blue

**FIGURE 3:**
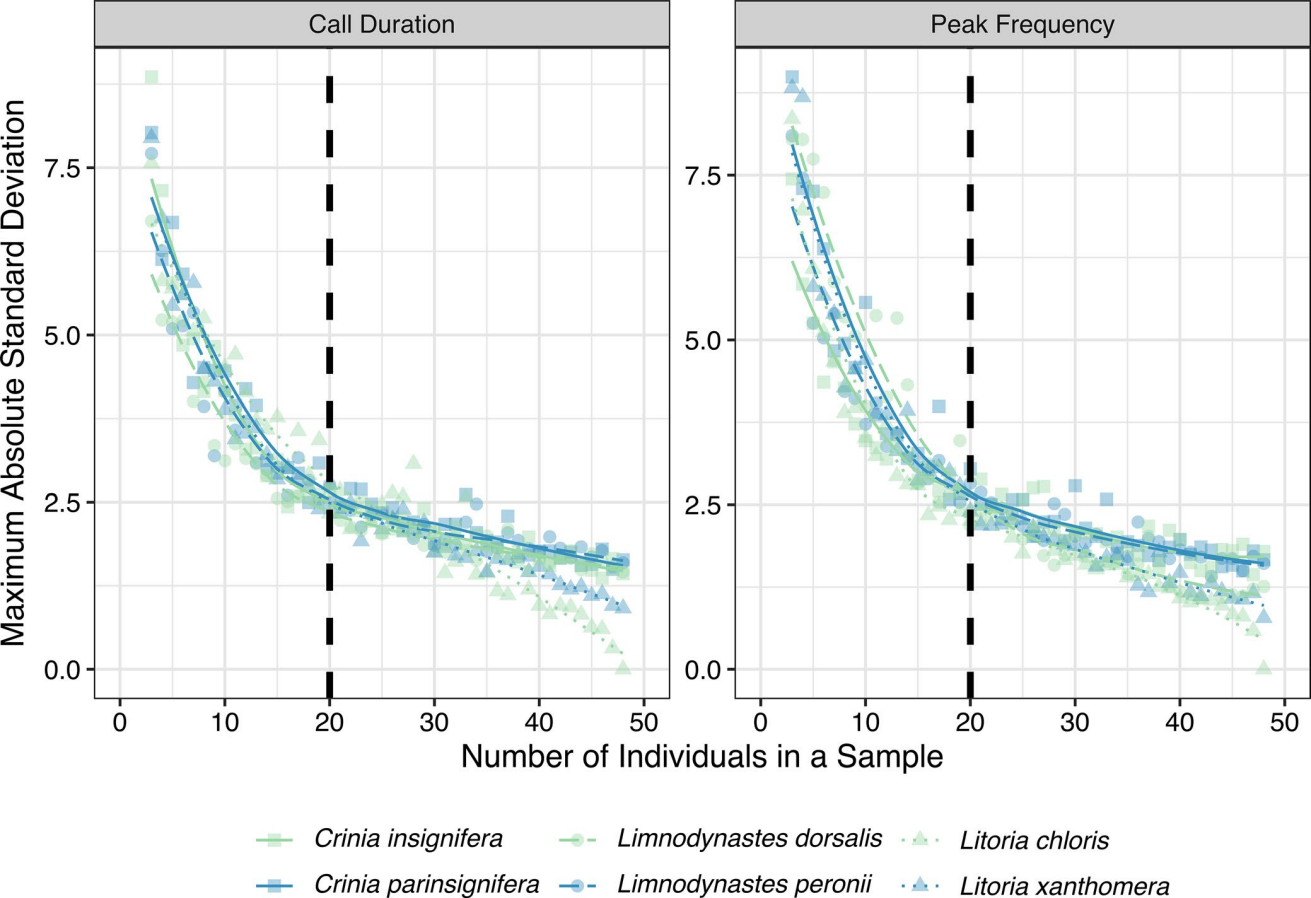
As the number of individuals measured increases, deviation of measured variance from actual variance decreases. Shapes and line types denote genera: *Crinia* as squares with solid lines, *Limnodynastes* as circles with dashed lines, and *Litoria* as triangles with dotted lines. Colors distinguish species within each genus: green versus blue.

Of the 1,487 individuals with recordings that met our filtering criteria, 762 were deemed of sufficient quality for inclusion in our analyses (Table 1). Call duration and peak frequency measurements of individuals clustered by species, but there was a great deal of variation within each intraspecific cluster (Figure 2; Table 1). We found that as the number of individuals in a sample increased, the sample more accurately represented the variability in call characteristics present in the population. Deviation from true population variability decreased as the number of individuals measured increased (Figure 3). Deviation appears to be greatest and decrease the most between 0 and 20 individuals sampled, with a less drastic decrease after including approximately 20 individuals, and this pattern was similar among all six species (Figure 3).

**FIGURE 4:**
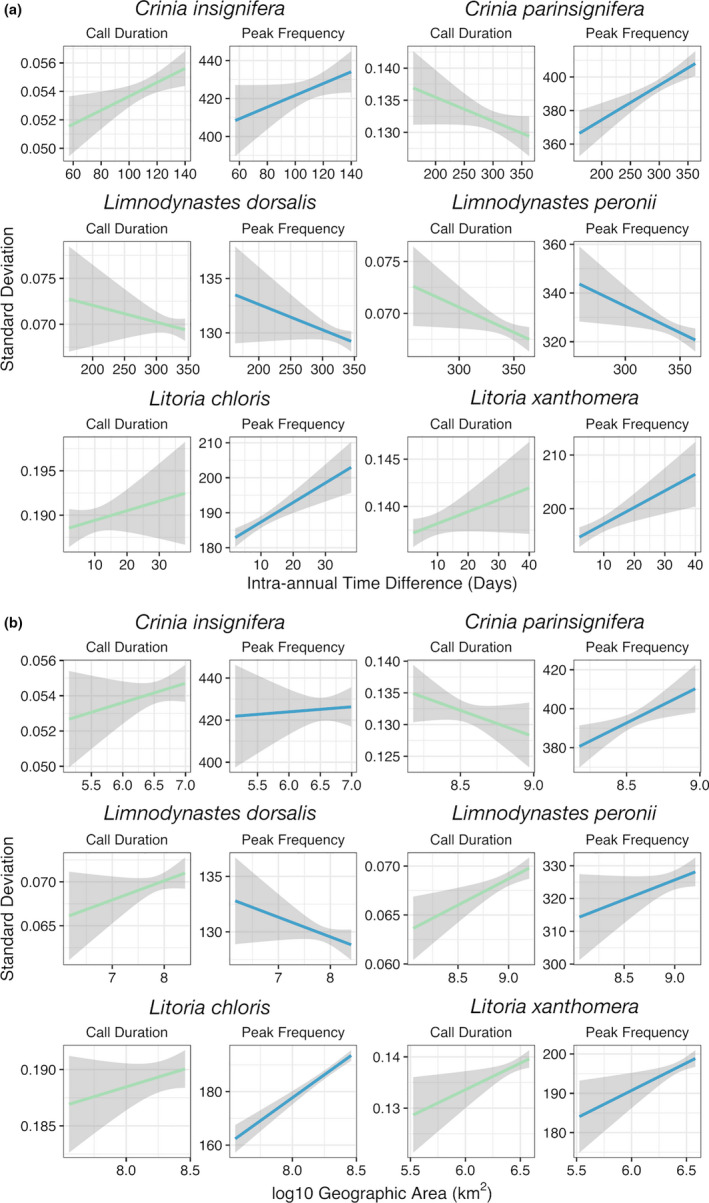
Vocal variability correlates with (a) intra‐annual time difference and (b) geographic area. Shaded area around each linear model represents standard error, which varies based on number of individuals within a given section of the model. Colors distinguish models for call duration (green) versus peak frequency (blue)

We found strong correlations of the variability of call duration and peak frequency with geographic area encompassed by and the number of days between individuals in a sample (Figure 4). Across species (i.e., using species as a random effect), the variability of call duration was positively correlated with geographic area and intra‐annual time difference. This relationship was statistically significant for geographic area (GLMM, estimate = .0418, *t* = 3.247, *df* = 5,998, *p*‐value = .001), but nonsignificant for intra‐annual time difference (GLMM, estimate = .0018, *t* = .142, *df* = 5,998, *p*‐value = .887). The variability of peak frequency was positively correlated with and statistically significant for both geographic area (GLMM, estimate = .0822, *t* = 6.39, *df* = 5,998, *p*‐value < .0001) and difference in days (GLMM, estimate = .0501, *t* = 3.88, *df* = 5,998, *p*‐value = .0001)

**FIGURE 5:**
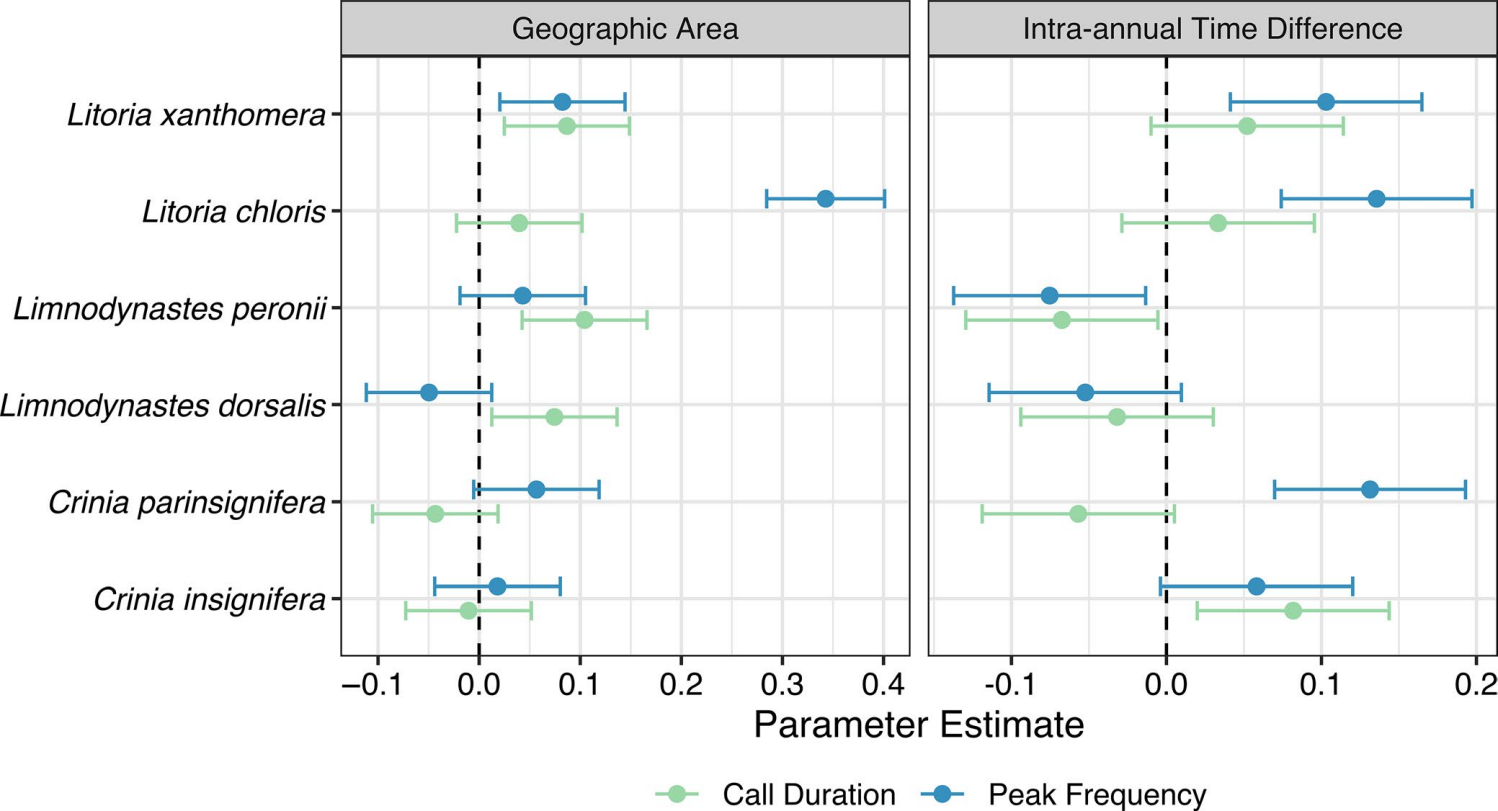
Correlation coefficients for call duration and peak frequency as a function of intra‐annual time difference and geographic area. Error bars represent 95% confidence intervals. Colors distinguish model estimates for call duration (green) versus peak frequency (blue)

When species were considered separately, the trends in variability of call duration and peak frequency were not the same across species (Table 2, Figures 4 and 5). Variability in call duration increased as geographic area increased for all species except *C*. *parinsignifera*. Variability in call duration increased as intra‐annual time difference increased for *C*. *insignifera*, *Lit*. *chloris*, and *Lit*. *xanthomera*, but decreased for *C*. *parinsignifera*, *Lim*. *dorsalis*, and *Lim*. *peronii*. Variability in peak frequency increased as the geographic area increased for *C*. *parinsignifera*, *Lim*. *peronii*, *Lit*. *chloris*, and *Lit*. *xanthomera*, but decreased for *Lim*. *dorsalis*, and showed no clear trend for *C*. *insignifera*. Variability in peak frequency in relation to intra‐annual time difference appeared to differ among genera, but not within species pairs: It increased as intra‐annual time difference increased for species in the *Crinia* and *Litoria* genera, but decreased for species in the *Limnodynastes* genus.

**TABLE 2:** Summary statistics for species‐specific generalized linear models

Species	Predictor variable	Dependent variable	Estimate	*t*‐statistic	*df*	*p*‐value
*Crinia insignifera*	Area	Call duration	−0.01050	−0.332	2	.7403
		Peak frequency	0.01813	0.573	2	.5668
	Days	Call duration	0.08177	2.592	2	.0097
		Peak frequency	0.05816	1.840	2	.0660
*Crinia parinsignifera*	Area	Call duration	−0.04339	−1.372	2	.1704
		Peak frequency	0.05662	1.792	2	.0735
	Days	Call duration	−0.05686	−1.799	2	.0723
		Peak frequency	0.13137	4.186	2	<.0001
*Limnodynastes dorsalis*	Area	Call duration	0.07446	2.359	2	.0185
		Peak frequency	−0.04959	−1.568	2	.1171
	Days	Call duration	−0.03186	−1.007	2	.3142
		Peak frequency	−0.05238	−1.657	2	.0978
*Limnodynastes peronii*	Area	Call duration	0.10425	3.311	2	.0010
		Peak frequency	0.04311	1.363	2	.1731
	Days	Call duration	−0.06749	−2.137	2	.0328
		Peak frequency	−0.07535	−2.387	2	.0172
*Litoria chloris*	Area	Call duration	0.03968	1.254	2	.2100
		Peak frequency	0.34275	11.526	2	<.0001
	Days	Call duration	0.03333	1.053	2	.2924
		Peak frequency	0.13560	4.324	2	<.0001
*Litoria xanthomera*	Area	Call duration	0.08680	2.753	2	.0060
		Peak frequency	0.08237	2.611	2	.0092
	Days	Call duration	0.05209	1.648	2	.0997
		Peak frequency	0.10302	3.272	2	.0011

Note

Area refers to geographic area (km^2^) of the convex hull encompassed by individuals in a sample. Days refers to the intra‐annual time difference (days) between individuals in a sample.

A species' range size did not correlate with the relationships of call duration as a function of geographic area (GLM, estimate = −1.5e‐8, *t* = −.264, *df* = 5, *p*‐value = .805), peak frequency as a function of geographic area (GLM, estimate = −3.2e‐8, t = −.242, *df* = 5, *p*‐value = .821), or peak frequency as a function of intra‐annual time difference (GLM, estimate = −4.1e‐8, *t* = −.45, *df* = 5, *p*‐value = .676), but it did significantly correlate with the relationship of call duration as a function of intra‐annual time difference (GLM, estimate = −1.1e‐7, *t* = −4.05, *df* = 5, *p*‐value = .015). The maximum male SVL for each species correlated with the relationship of call duration as a function of geographic area (GLM, estimate = .0026, *t* = 4.85, *df* = 5, *p*‐value = .0083). A species' maximum SVL did not correlate with the relationships of peak frequency as a function of geographic area (GLM, estimate = .0013, *t* = .417, *df* = 5, *p*‐value = .698), call duration as a function of intra‐annual time difference (GLM, estimate = −6.8e‐4, *t* = −.468, *df* = 5, *p*‐value = .664), or peak frequency as a function of intra‐annual time difference (GLM, estimate = −.0023, *t* = −1.203, *df* = 5, *p*‐value = .295).

## 4 DISCUSSION

We leveraged a unique continental‐scale citizen science dataset to quantify the variability in call duration and peak frequency across broad spatial and temporal scales. Although only half of all FrogID recordings we examined were used in our study, the dataset gave us a remarkable degree of spatial and temporal representation. Among only the calls we analyzed, recordings were distributed throughout most of each species' geographic range (Figure S1) and spanned the duration of the species' breeding seasons over the 2 year study period (Table 1). FrogID recordings which did not meet our quality standards still remain important biodiversity records and may be suitable for other bioacoustic studies.

For six frog species across two families and three genera, we found high intraspecific variability of call duration and peak frequency (Figure 2). Our results highlight the importance of considering the number of individuals analyzed when examining vocalizations: a sample size of less than 20 individuals is unlikely to capture the true variability in a species' advertisement call (Figure 3). Male advertisement calls are often used to delineate and describe species as new to science, but the number of individuals analyzed can be relatively small, sometimes only 1–5 individuals (Matsui, 1997; Pombal et al., 1995; Roberto et al., 2017). We recommend that researchers analyze advertisement calls from at least 20 individuals per clade or taxon (Martins & Jim, 2004). Although our recommendation is based on only six species, the geographic and taxonomic scope of our study was relatively broad. We encourage future studies to test whether this trend holds true in other regions and taxonomic groups. We acknowledge that 20 or more recordings may be logistically challenging, especially for rarely detected species in remote locations, but future call descriptions should strive to sample 20 individuals, captured over time and space, to truly describe a species' vocalization.

Call duration and peak frequency both varied across time and space for all six species. We found that variability of both call duration and peak frequency was positively correlated with both geographic area encompassed by locations of individuals and intra‐annual time difference between individuals in a sample. When we considered each species separately, this trend was relatively consistent.

As the intra‐annual time difference between individuals in a sample increased, the vocalizations of those individuals became more variable (Figures 4 and 5). This correlation could be related to changes in individuals throughout the breeding season, such as age, body size, hormone fluctuations, or vocal maturation as the breeding season progresses (Ryan, 2001; Wells, 2007). While some studies have investigated temporal patterns, it is unclear whether call variability does (Smith & Hunter, 2005) or does not (Gambale et al., 2014; Giacoma et al., 1997) have temporal trends. Regardless of the reason for intra‐annual variability in call characteristics, our findings highlight the importance of considering time of year when measuring frog vocalizations.

As the geographic area among individuals increased, their vocalizations also became more variable (Figures 4 and 5). These results suggest that vocalizations follow an isolation by distance model, which has also been suggested by previous studies of advertisement calls (Marova et al., 2010; Rafiński & Babik, 2000; Ryan et al., 1996). Generally, there is strong evidence for spatial, population‐based differences in call characteristics (Baraquet et al., 2015; Capranica et al., 1973; Hernández‐Herrera & Pérez‐Mendoza, 2020; Jang et al., 2011; Littlejohn & Roberts, 1975; Ohmer et al., 2009; Rodríguez et al., 2010). In addition to the phenotypic gradient represented across an isolation by distance model, there are geographic barriers to gene flow like mountain ranges, ocean divides, and cities, which separate groups and may lead to vocal divergence (Jang et al., 2011; Klymus et al., 2012). Our findings emphasize the need to measure vocalizations across a species' entire geographic range to encompass its call variability.

While variability in both call duration and peak frequency most often had positive correlations with time and space, this relationship was not true for every species we analyzed. In fact, several relationships had strong negative correlations (e.g., *C*. *parinsignifera*, *Lim*. *dorsalis*, and *Lim. peronii* (Figure 4a); *C*. *parinsignifera* and *Lim*. *dorsalis* (Figure 4b)). We are unsure what may drive the negative correlations we observed. One potential driver is character displacement (Brown & Wilson, 1956; Jang & Gerhardt, 2006), so that within a species, individuals from the same population alter their calls when overlapping in time or space to distinguish themselves from their neighbors when competing to attract a mate. This pattern of sympatric character displacement has been observed between bird species (Kirschel et al., 2009; Wallin, 1986), but has yet to be studied in frog species. One study suggests that *C*. *parinsignifera* and *Lim*. *dorsalis* have “tuned hearing” that only picks up the specific frequency of conspecific calls because these species are often found in multi‐species choruses (Loftus‐Hills & Johnstone, 1970). Tuned hearing may explain the negative trends we observed because although small vocal variation may occur in these species, over more space or time, their calls would be constrained by their hearing, resulting in less variation in broader spatial or temporal scales than at smaller scales. Alternatively, breeding season is unlikely to play a role, as *C*. *insignifera* and *Lim*. *dorsalis* breed in the winter, *Lit*. *chloris*, *Lit*. *xanthomera*, and *Lim*. *peronii* breed in the summer, and *C*. *parinsignifera* breeds year‐round (Barker et al., 1995). Similarly, call structure was unlikely to be responsible, as it was relatively consistent within each genus studied. Further, we are unsure of the role of plasticity, which could potentially contribute to either positive or negative correlations observed (Price et al., 2003). Roles of these and other factors should be discerned by future research to determine the mechanisms resulting in the observed patterns.

Each species has a unique life history, so each species is likely affected differently and to varying degrees by the ecological and evolutionary drivers of vocal variation. We investigated species' range size as a potential reason for the interspecific differences across correlations, but found little evidence that range size influenced the relationships of either call characteristics as a function of space or time. While we did find evidence suggesting that a larger range size could lead to more variable call duration over time, overall, our findings suggest that a species' range size does not determine the vocal variation possible across its range or throughout time. We also considered whether body size had a relationship with advertisement call variability. Studies show that vocalizations vary among individuals and among species based on body size (Blair, 1964; McClelland et al., 1996; Rodríguez et al., 2010). It was previously unknown whether a larger maximum body size enables more vocal variability within a species, and we found a correlation between maximum male SVL of a species and the relationship of call duration as a function of geographic area. However, most body size correlations were weak and nonsignificant. Other factors likely to influence the species‐specific relationships observed include ecological considerations such as weather, habitat type, elevation, or anthropogenic effects, and evolutionary constraints such as call complexity, vocal repertoire, and morphology (Ryan, 2001; Wells, 2007). Every species is likely to respond to these factors differently, which leads to great potential vocal diversity within some species, as well as potential species divergence. The distinction between intraspecific diversity and speciation is yet to be determined, although we are confident in the species delineations for those analyzed in this study.

Ultimately, our findings reveal strong temporal and spatial patterns of frog vocalizations. However, several limitations of this study should be improved upon in future studies. Due to our macroecological approach and reliance on citizen science recordings, we cannot ensure every recording is a unique individual. To increase this likelihood, we filtered recordings by location. These methods produced meaningful results for our investigation of variability among individuals, but we suggest additional studies also investigate call variation within individuals of a specific locale over time. We were also unable to rule out whether temperature or body size plays a role in the patterns we present due to their influence at the individual level (Cocroft & Ryan, 1995; McClelland et al., 1996; Wilczynski et al., 1993). Frogs also modify their calls depending on social context (Hernández‐Herrera & Pérez‐Mendoza, 2020), but we were unable to incorporate information about whether frogs were calling solitarily or as part of a chorus. Rather, we filtered the recordings we used to those with only the target species calling and minimum background noise, which eliminated most chorus calling from our analyses. Future studies could incorporate this variable, but should be aware of the trade‐offs between citizen science data volume and scientific specificity. For example, to address social context using our dataset would have required post hoc determination of singularity versus chorus and estimations of chorus size based on audio interpretation. Finally, we measure intra‐annual time difference as the maximum range of days among individuals in a sample. This measure currently does not account for the distribution of individuals in the random sample in regard to the breeding season (i.e., more individuals could be sampled closer to one end of the calling season than the other end), potentially limiting our analyses related to intra‐annual time difference. Future work should focus on testing our observed patterns in other species, both within related genera and across various lineages. Testing the patterns we present at several phylogenetic levels would help to determine whether phylogeny or environment has the greatest influence on call variability (Bosch & De la Riva, 2004; Erdtmann & Amézquita, 2009; Welch et al., 2014). Lastly, we used two key vocal characteristics (i.e., call duration and peak frequency), but other bioacoustic variables may be valuable. Our approach could be generalized to include these additional call characteristics, as well as more species, in different habitats, and from other continents.

While many studies have tested spatial patterns of vocal variability (Baraquet et al., 2015; Capranica et al., 1973; Jang et al., 2011; Littlejohn & Roberts, 1975; Rodríguez et al., 2010; Ryan et al., 1996), few have tested temporal patterns (Gambale et al., 2014; Giacoma et al., 1997; Smith & Hunter, 2005). Our results highlight the value of using citizen science data to assess the patterns of acoustic or morphological variability at scales previously not possible. We clearly highlight the inherent variability in advertisement calls, which should be accounted for in future bioacoustic studies. Comparisons of frog calls and descriptions of new species that only measure a few individuals from a single locale at a single point in time likely fail to properly capture the variability that exists within a species' vocalization. In order to accurately understand anuran accents, the number of individuals in a sample must be numerous (*N* ≥ 20), encompass a large geographic area relative to the species range, and be collected throughout its calling season. Citizen science will continue to play a role enabling such studies, and coupled with targeted fieldwork, could supply ecologists with increasingly robust datasets to find and explore nuances in similar macroecological patterns.

## CONFLICT OF INTERESTS

The authors report no conflicts of interest.

## AUTHOR CONTRIBUTION

**Savannah J. Weaver:** Data curation (equal); Formal analysis (equal); Visualization (equal); Writing‐original draft (equal); Writing‐review & editing (equal). **Corey T. Callaghan:** Conceptualization (equal); Formal analysis (equal); Methodology (equal); Visualization (equal); Writing‐original draft (equal); Writing‐review & editing (equal). **Jodi J. L. Rowley:** Conceptualization (equal); Visualization (equal); Writing‐original draft (equal); Writing‐review & editing (equal).

### OPEN RESEARCH BADGES

This article has been awarded Open Data and Open Materials Badges. All materials and data are publicly accessible via the Open Science Framework at https://github.com/science-with-sav/anuran_accents_data_code.

## Supporting information

Fig S1: Geographic range for each species (shaded area) and location of each FrogID recording (black circles) used in our analyses. Horizontal axis is longitude. Vertical axis is latitude

## Data Availability

Figures and analyses were completed in R version 3.6.3 (R Core Team, 2020) and relied heavily on the tidyverse workflow (Wickham, 2017). Data and annotated code are archived in Zenodo (https://doi.org/10.5281/zenodo.4016911).
